# Neutrophil Extracellular Traps in the Tumor Microenvironment: Mechanisms, Immune Regulation, and Implications for Immune Checkpoint Inhibitor Therapy

**DOI:** 10.3390/ijms27188278

**Published:** 2026-09-17

**Authors:** Chung-Wen Kuo, Hsin-Ying Clair Chiou, Shih-Yu Huang, Chia-Che Wu, Yu-Li Su

**Affiliations:** 1 Division of Hematology Oncology, Department of Internal Medicine, Kaohsiung Chang Gung Memorial Hospital, Chang Gung University College of Medicine, Kaohsiung 83301, Taiwan; bulakuo@gmail.com (C.-W.K.);; 2 Genomic & Proteomic Core Laboratory, Department of Medical Research, Kaohsiung Chang Gung Memorial Hospital, Kaohsiung 83301, Taiwan; 3 Department of Applied Chemistry, National Chi Nan University, Nantou 54561, Taiwan; 4 Doctoral Program of Clinical and Experimental Medicine, College of Medicine, National Sun Yat-sen University, Kaohsiung 804201, Taiwan; 5 School of Medicine, College of Medicine, National Sun Yat-sen University, Kaohsiung 804201, Taiwan; 6 Institute for Translational Research in Biomedicine, Kaohsiung Chang Gung Memorial Hospital, Kaohsiung 83301, Taiwan; 7 Cancer Center, Kaohsiung Chang Gung Memorial Hospital, Kaohsiung 83301, Taiwan

**Keywords:** neutrophil extracellular traps, NETosis, tumor microenvironment, immune checkpoint inhibitors, cancer immunotherapy, tumor-associated neutrophils

## Abstract

Neutrophil extracellular trap formation (NETosis) has emerged as a pivotal regulator of the tumor immune microenvironment and a key determinant of response to cancer immunotherapy. NETs—web-like chromatin fibers decorated with histones, citrullinated histone H3, myeloperoxidase, neutrophil elastase, and matrix metalloproteinase-9—are induced by tumor-derived signals through classical lytic, vital non-lytic, and gasdermin D-dependent pathways, and exert context-dependent, predominantly pro-tumorigenic effects. Within the tumor microenvironment, NETs remodel the stroma, suppress CD8^+^ T-cell, natural killer-cell, and dendritic-cell function, promote regulatory T-cell and M2 macrophage polarization, and physically shield tumor cells from cytotoxic attack. By carrying programmed death-ligand 1 and driving T-cell exhaustion and immune exclusion, NETs compromise the efficacy of immune checkpoint inhibitors (ICIs) targeting PD-1/PD-L1, CTLA-4, and next-generation checkpoints, as well as adoptive cell therapies. This review synthesizes the molecular mechanisms of NETosis, its multifaceted immunomodulatory roles, and emerging NET-derived biomarkers—including MPO-DNA and citrullinated histone H3-DNA complexes—that may predict immunotherapy response and toxicity. We further appraise therapeutic strategies, including PAD4 inhibitors, DNase I, CXCR2 antagonists, and nanoparticle-based platforms, and underscore the need for standardized NET quantification, careful preservation of host defense, and biomarker-guided clinical trials to translate NET modulation into durable improvements in immunotherapy outcomes.

## 1. Introduction

Neutrophil extracellular trap (NET) formation encompasses cellular processes that lead to the formation and extracellular release of NETs and may occur with or without neutrophil lysis [1]. The term “NETosis” is commonly used to describe these processes, although its usage and definition remain subject to ongoing nomenclature debate [2]. These web-like structures, composed of DNA fragments decorated with histones and granular proteins such as citrullinated histone H3 (CitH3), myeloperoxidase (MPO), neutrophil elastase (NE), cathepsin G (CG) and matrix metalloproteinase 9 (MMP-9) [3], enable neutrophils to capture and eliminate pathogens, including bacteria, fungi, viruses and parasites [4,5,6]. Within the immune system, NETosis serves as a first-line innate defense mechanism by trapping and eliminating extracellular microbes, thereby limiting the spread of infection [7,8]. However, dysregulated NETosis can be detrimental to the host, contributing to autoimmune, cardiovascular, and inflammatory diseases, as well as promoting cancer progression through excessive NET formation [9].

Advances in immuno-oncology, particularly immune checkpoint blockade and cell-based therapies, have transformed the management of several solid and hematological malignancies by restoring or amplifying antitumor immune responses [10,11,12]. Nevertheless, primary and acquired resistance remain frequent, reflecting complex immune escape mechanisms within the tumor microenvironment (TME), including the contributions of tumor-associated neutrophils (TANs) and neutrophil extracellular traps (NETs) to immune exclusion and the functional exhaustion of cytotoxic lymphocytes [13]. Recent translational studies further indicate that tumor cell-released autophagosomes (TRAPs) activate the high-mobility group box 1 (HMGB1)-Toll-like receptor 4 (TLR4)-myeloid differentiation primary response 88 (MyD88)-extracellular signal-regulated kinase (ERK)/p38 mitogen-activated protein kinase (p38 MAPK) signaling axis to induce NET formation. These TRAP-induced NETs exhibit high levels of programmed death-ligand 1 (PD-L1), which directly suppresses T-cell function and contributes to the formation of pre-metastatic immunosuppressive niches in a breast cancer pulmonary metastasis model [14]. In addition, next-generation immune checkpoints, such as T cell immunoreceptor with Ig and ITIM domains (TIGIT), lymphocyte-activation gene 3 (LAG-3) and T-cell immunoglobulin and mucin-domain-containing protein 3 (TIM-3), are emerging as key partners for PD-1/PD-L1 and CTLA-4 blockade [15], with tumor cells and myeloid-derived suppressor cells (MDSCs) expressing ligands for these receptors (e.g., galectin-9, fibrinogen-like protein 1 [FGL1] and CD155/CD112) to promote immune evasion and contribute to primary resistance to immune checkpoint inhibitors (ICIs) [16,17,18].

The clinical relevance of NETosis in oncology is supported by mounting evidence across multiple tumor types. In hepatocellular carcinoma (HCC), high intratumoral neutrophil density is an independent predictor of poor recurrence-free and overall survival [19]. This observation is further supported by a meta-analysis of 20 studies involving 3946 patients, which reported pooled hazard ratios of 1.68 for recurrence-/disease-free survival (RFS/DFS) and 1.66 for overall survival (OS) in patients with high intratumoral neutrophil infiltration [20]. In pancreatic ductal adenocarcinoma (PDAC), NETs promote a procoagulant and prothrombotic state, and pharmacological inhibition of NET formation attenuates hypercoagulability in preclinical models [21]. Collectively, these observations suggest that NETosis-related markers may represent promising but still preliminary prognostic indicators, warranting prospective, clinical validation in additional neutrophil-rich tumor types such as non-small cell lung cancer (NSCLC) and colorectal cancer (CRC) [22,23]. Direct NET-specific prognostic data, though still limited, are also beginning to emerge. Elevated circulating CitH3 independently predicts short-term mortality in patients with advanced cancer [24] and overall mortality in a prospective cohort of 957 cancer patients [25], while stromal NET density assessed by MPO/CitH3 dual immunofluorescence has been identified as an independent prognostic factor for recurrence in cervical cancer [26].

NETs demonstrate context-dependent pro- and antitumor effects, functioning as a “double-edged sword” in cancer biology [27]. On the one hand, several NET-associated components have been reported to exert direct cytotoxic effect against tumor cells. Extracellular histones disrupt cellular membranes and induce dose-dependent cytotoxicity. While this effect has been established in epithelial and endothelial cells, emerging evidence suggests that tumor cells may also be susceptible [28]. Likewise, reactive oxygen species (ROS) generated by NADPH oxidase (NOX) during NETosis may impair the function and viability of adjacent cells, including cytotoxic lymphocytes, while also inducing oxidative DNA damage and redox imbalance in neighboring cancer cells [29]. In addition, NET-derived DNA has been proposed to activate the cyclic GMP-AMP synthase-stimulator of interferon genes (cGAS-STING) pathway, thereby promoting type I interferon production and potentially facilitating dendritic cell-mediated cross-priming of CD8^+^ T cells, which may contribute to the initiation of adaptive antitumor immunity [30]. On the other hand, in established tumors with immunosuppressive TMEs, accumulating evidence suggests that this balance shifts toward NET-mediated immune exclusion, extracellular matrix (ECM) remodeling, and the facilitation of metastasis [13]. This functional dichotomy appears to be determined by tumor histological subtype, disease stage, the local cytokine milieu, and the NET composition, highlighting the need to account for these context-dependent factors when designing NET-targeted therapeutic strategies.

Although NETs are now well established as drivers of tumor growth, invasion, metastasis, and treatment resistance, their precise impact on antitumor immunity and the efficacy of modern immunotherapies remains incompletely defined. Therefore, the primary aim of this review is to synthesize current knowledge on how NETosis shapes the TME, delineate its influence on the efficacy of ICIs as well as other immunotherapeutic strategies, and evaluate the therapeutic potential of pharmacologically targeting NETosis to improve clinical outcomes.

## 2. Mechanisms of NETosis

### 2.1. Classification and Formation of NETs

NETs consist of decondensed chromatin fibers decorated with histones and granular or cytosolic proteins that are released into the extracellular space following distinct neutrophil activation programs [31,32]. A broad spectrum of stimuli, including microbial products, damage-associated molecular patterns (DAMPs), cytokines, chemokines, immune complexes, platelets, tumor-derived factors, and metabolites, can engage pattern-recognition or chemokine receptors on neutrophils and trigger intracellular signaling cascades culminating in NET release [8,33].

It should be noted that the term “NETosis” is used throughout this review, consistent with prevailing convention, to encompass all processes culminating in NET release; however, this terminology remains a subject of ongoing debate, as it was originally coined to describe a terminal, lytic form of neutrophil death and does not readily accommodate the vital, non-lytic pathway in which neutrophils remain viable. Where distinction is scientifically important, this review uses the terms “classical (lytic) NETosis”, equivalent to what is termed “suicidal NETosis” in other classification schemes, and “vital (non-lytic) NETosis” to avoid conflating NET release with an obligatory cell-death outcome [34].

**Figure 1:**
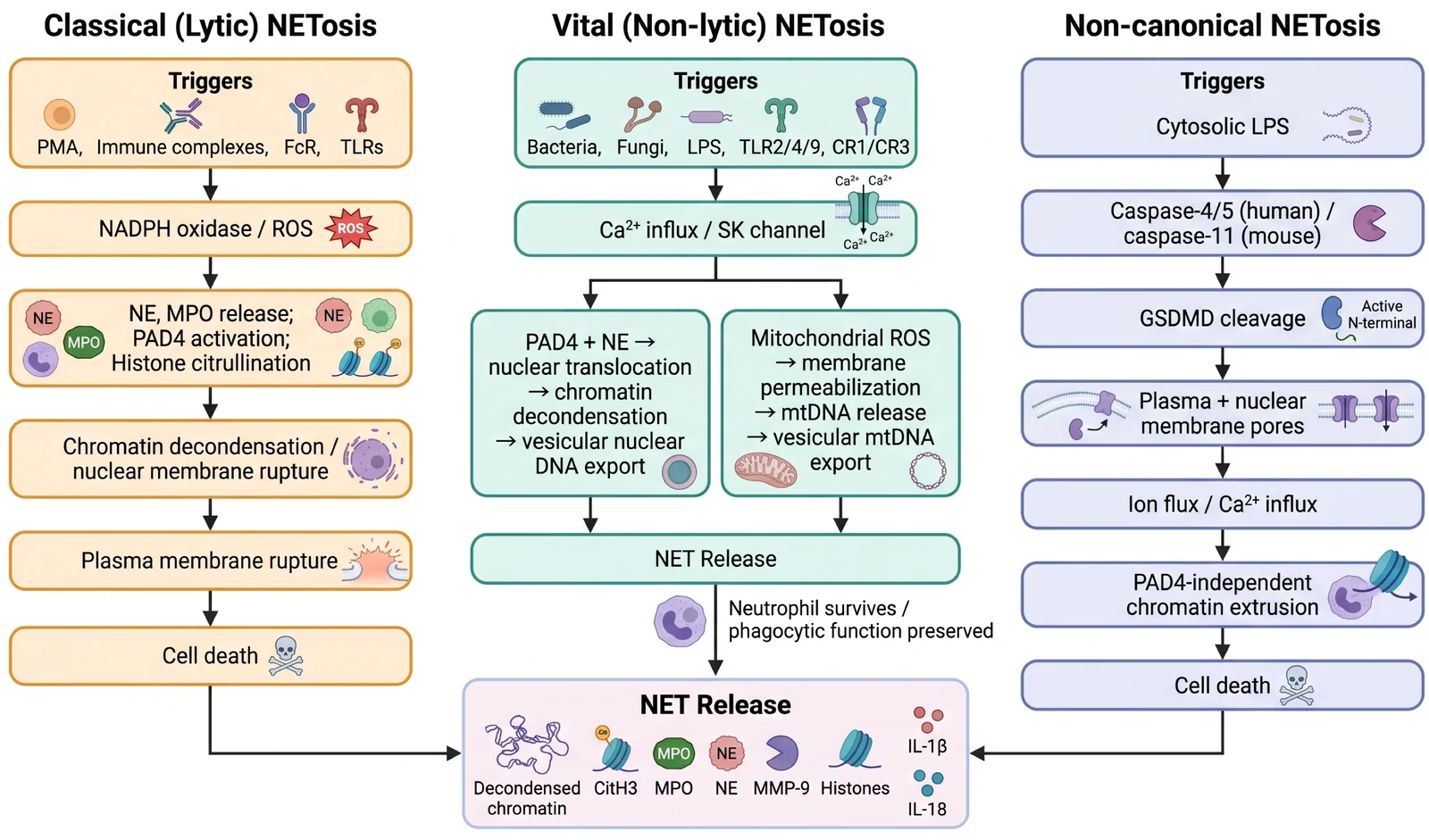
Mechanistically distinct pathways of NETosis. Three major forms of neutrophil extracellular trap (NET) formation are illustrated. Classical (lytic) NETosis is triggered by phorbol myristate acetate (PMA), immune complexes, and activation of Fc receptors (FcRs) or Toll-like receptors (TLRs), leading to NADPH oxidase (NOX)-dependent reactive oxygen species (ROS) generation, neutrophil elastase (NE) and myeloperoxidase (MPO) release, peptidyl arginine deiminase 4 (PAD4)-mediated histone citrullination, chromatin decondensation, nuclear membrane rupture, and terminal neutrophil death. Vital (non-lytic) NETosis is induced by microbial stimuli through TLRs and complement receptors (CR1/CR3), resulting in calcium influx, PAD4 and NE activation, chromatin decondensation, and vesicular export of nuclear or mitochondrial DNA (mtDNA), while preserving neutrophil viability and phagocytic function. Non-canonical NETosis is initiated by cytosolic lipopolysaccharide (LPS), which activates caspase-4/5 in humans, leading to gasdermin D (GSDMD) cleavage, pore formation, ion flux, and PAD4-independent chromatin extrusion followed by cell death. Despite distinct upstream signaling mechanisms, all three pathways converge on the release of extracellular chromatin decorated with citrullinated histone H3 (CitH3), MPO, NE, matrix metalloproteinase-9 (MMP-9), histones, and inflammatory mediators, including interleukin-1β (IL-1β) and interleukin-18 (IL-18), which together constitute the NET scaffold. Figure is created in BioRender. Su, Y. (2026) https://BioRender.com/4uae1kh.

Current evidence supports three principal categories of NETosis, classified according to their triggering mechanisms, ROS dependent and cell fate outcome (Figure 1): (i) classical (lytic) NETosis, a ROS- and PAD4-dependent terminal pathway resulting in neutrophil death; (ii) vital (non-lytic) NETosis, a rapid process involving the release of nuclear or mitochondrial DNA (mtDNA) while neutrophils remain viable and retain effector functions; and (iii) non-canonical NETosis, driven by gasdermin D (GSDMD) downstream of inflammasome activation, independently of the NOX-ROS axis. In addition, some classification schemes formally designate mitochondrial NETosis, in which neutrophils release mitochondrial rather than nuclear DNA, as a distinct category [9,34]. However, because this pathway shares the defining features of vital NETosis such as membrane integrity preserved, vesicular export, neutrophil survival, and is primarily distinguished by DNA source rather than by a fundamentally distinct triggering mechanism, it is discussed in this review as a DNA-source variant within the vital NETosis framework (Section 2.4).

Classical NETosis is initiated when stimuli such as phorbol 12-myristate 13-acetate (PMA) or immune complexes engage Fc receptors (FcRs) and Toll-like receptors (TLRs) on the neutrophil surface, triggering assembly of the NADPH oxidase complex and generation of ROS [35,36]. ROS accumulation drives two parallel processes that converge on chromatin decondensation. On one branch, ROS, together with an increase in cytosolic Ca^2+^, directly activates PAD4. Activated PAD4 translocates to the nucleus and mediates histone hypercitrullination [35,36]. On the other, largely PAD4-independent branch, neutrophil elastase (NE) and myeloperoxidase (MPO) are released from the azurophilic granules (azurosomes) and translocate to the nucleus, where NE partially degrades histones while MPO synergizes with NE to further promote chromatin decondensation independently of its enzymatic activity [35,36]. These two convergent processes jointly drive progressive chromatin decondensation and nuclear envelope rupture, followed by plasma membrane disintegration and extrusion of DNA–protein networks (Figure 1). Due to its dependence on sustained ROS production and progressive membrane disintegration, classical NETosis typically occurs over 2–4 h and represents an irreversible terminal fate for neutrophils.

In contrast, vital NETosis can be triggered within minutes by exposure to bacteria, fungi or lipopolysaccharide (LPS), through engagement of TLR2, TLR4 and TLR9, as well as complement receptors 1 and 3 (CR1/3) [37,38]. Receptor activation induces small-conductance calcium-activated potassium (SK) channel-mediated Ca^2+^ influx, promoting the translocation of PAD4 and NE to the nucleus. Chromatin decondensation occurs without disruption of the plasma membrane, and nuclear or mtDNA is subsequently packaged and exported via vesicular transport rather than membrane rupture [39,40] (Figure 1). Because plasma membrane integrity is maintained, the resulting anucleate neutrophil remains viable and retains phagocytic and chemotactic functions, distinguishing vital NETosis from lytic pathways.

The non-canonical pathway is activated when LPS gains access to the cytosol and directly binds the CARD domain of caspase-4 and caspase-11, triggering their autoproteolytic activation independently of the canonical NLRP3 or AIM2 sensors. These caspases process GSDMD to release its pore-forming N-terminal fragment, which inserts into both the plasma and nuclear membranes independently of PAD4 [41,42]. Downstream potassium efflux through GSDMD pores can secondarily engage the NLRP3 inflammasome, amplifying caspase-1-dependent IL-1β/IL-18 maturation [43]. Pore formation permits ion flux and Ca^2+^ influx, driving nuclear swelling and chromatin extrusion, ultimately resulting in cell death and NET release through a mechanism distinct from classical lytic NETosis (Figure 1).

Despite their distinct triggers and kinetics, all three pathways converge on the extracellular release of chromatin structures decorated with CitH3, MPO, NE, matrix metalloproteinase-9 (MMP-9), and pro-inflammatory cytokines such as interleukin-1β (IL-1β) and IL-18 (Figure 1). Recent mechanistic studies further indicate that tumor cell-released autophagosomes (TRAPs), as well as chemotherapy- or radiotherapy-induced DAMPs signaling through HMGB1-TLR4, can amplify NOX-dependent ROS production and PAD4 activity via p38/ERK signaling, thereby accelerating tumor-associated NETosis [14,44].

### 2.2. Key Signaling Pathways Inducing NETosis

Multiple converging signaling pathways regulate NETosis, including MAPK cascades [8], calcium-dependent PAD4 activation, and cell cycle-associated cyclin-dependent kinases 4 and 6 (CDK4/6), which modulate chromatin organization and cytoskeletal dynamics [45]. Engagement of Toll-like receptors (TLRs), Fc receptors (FcRs), complement receptors, and chemokine receptors couple diverse extracellular stimuli to NOX activation and ROS production. These events subsequently promote NE and MPO translocation, histone modification, and chromatin decondensation [32,46].

Non-lytic NETosis may occur through either ROS-dependent or ROS-independent pathway and can be triggered by platelet TLR4 signaling, the C3-C3a receptor (C3aR) pathway, or TLR2 activation by specific pathogens and endogenous ligands [47,48]. Beyond its central role in histone citrullination and NET formation, PAD4 has been shown to regulate C-X-C chemokine receptor 2 (CXCR2) expression, thereby linking neutrophil chemotaxis with NET generation [49].

### 2.3. Tumor-Specific Inducers of NETosis

**Figure 2:**
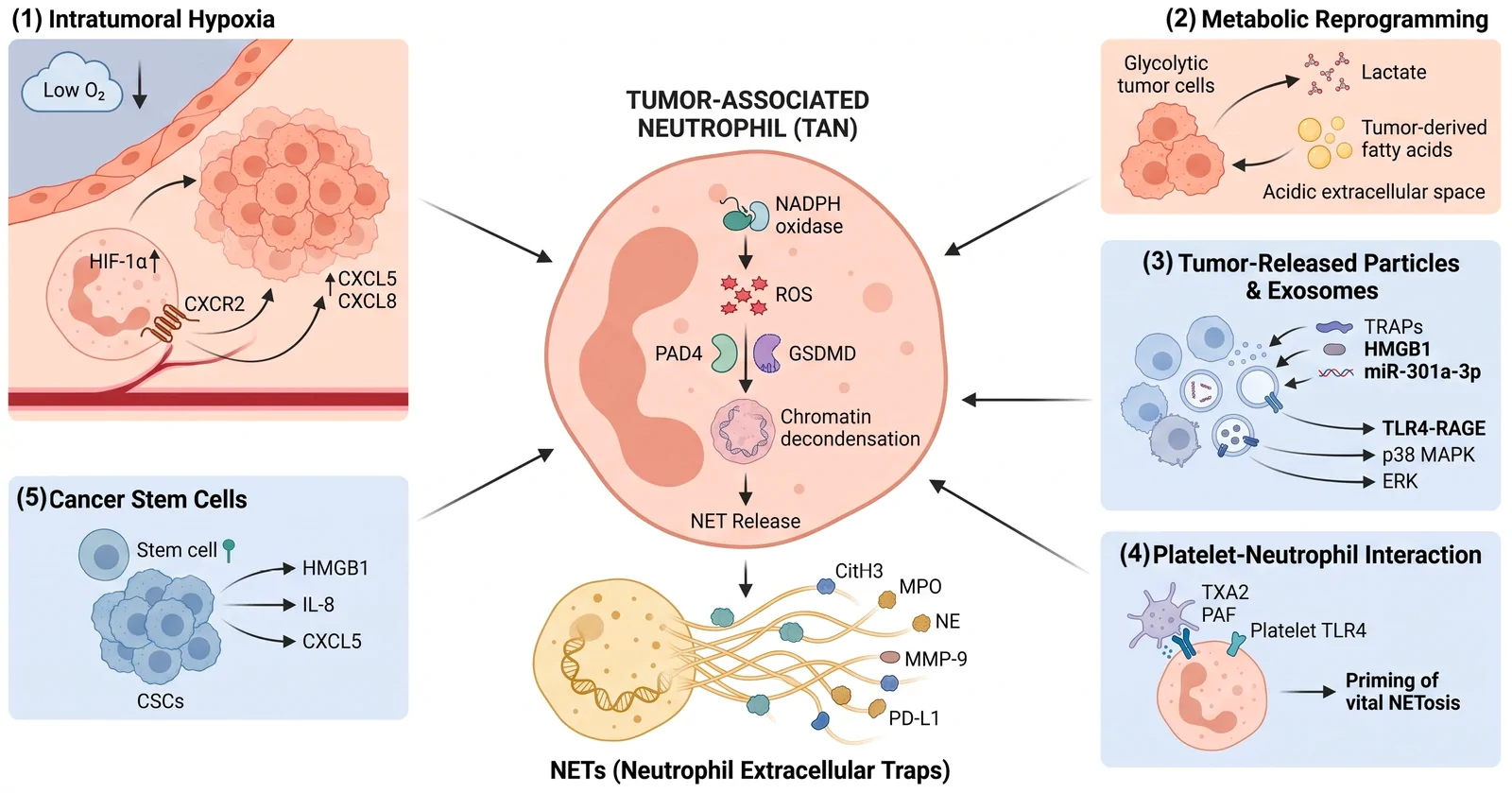
Tumor-specific inducers of NETosis within the tumor microenvironment (TME). Diverse tumor-derived and stromal signals converge on tumor-associated neutrophils (TANs) to promote neutrophil extracellular trap (NET) formation. Intratumoral hypoxia activates hypoxia-inducible factor 1α (HIF-1α) and C-X-C chemokine receptor 2 (CXCR2) signaling, accompanied by increased C-X-C motif chemokine ligand 5 (CXCL5) and CXCL8. Metabolic reprogramming further influences NETosis through lactate accumulation, tumor-derived fatty acids, and extracellular acidification. Tumor-released autophagosomes (TRAPs) and exosomes carrying high-mobility group box 1 (HMGB1) or microRNA-301a-3p (miR-301a-3p) activate Toll-like receptor 4 (TLR4), the receptor for advanced glycation end products (RAGE), p38 mitogen-activated protein kinase (p38 MAPK), and extracellular signal-regulated kinase (ERK) signaling. Platelet–neutrophil interactions mediated by thromboxane A2 (TXA2), platelet-activating factor (PAF), and platelet TLR4 prime vital NETosis, whereas cancer stem cells (CSCs) release HMGB1, interleukin-8 (IL-8), and CXCL5 to recruit and activate neutrophils. These signals converge on nicotinamide adenine dinucleotide phosphate (NADPH) oxidase-dependent reactive oxygen species (ROS) production, peptidyl arginine deiminase 4 (PAD4), and gasdermin D (GSDMD), leading to chromatin decondensation and the release of NETs decorated with citrullinated histone H3 (CitH3), myeloperoxidase (MPO), neutrophil elastase (NE), matrix metalloproteinase-9 (MMP-9), and programmed death-ligand 1 (PD-L1). Figure is created in BioRender. Su, Y. (2026) https://BioRender.com/sri33ej.

Beyond canonical stimuli, tumor-specific signals represent a distinct and clinically relevant class of NETosis inducers (Figure 2). Intratumoral hypoxia activates hypoxia-inducible factor 1α (HIF-1α) in infiltrating neutrophils, upregulating CXCR2 and increasing their responsiveness to tumor-derived chemokines, including C-X-C motif chemokine ligand 5 and 8 (CXCL5/8). This process promotes local NET formation independently of systemic inflammatory cues [46]. Tumor metabolic reprogramming further modulates NETosis. Under glucose-restricted conditions within the TME, immature neutrophil subsets shift toward mitochondrial fatty acid oxidation, thereby enhancing ROS production, sustaining NET formation, and promoting metastasis [50]. However, these metabolic effects are concentration- and context-dependent. Extracellular acidification, a hallmark of highly glycolytic and lactate-producing tumors, can suppress ROS-dependent NET release by impairing neutrophil glycolysis and oxidative burst, indicating that lactate and pH exert complex, rather than uniformly stimulatory, effects on NETosis [51].

Tumor-derived exosomes carrying oncogenic microRNAs, such as miR-301a-3p, or surface-associated proteins, such as HMGB1, engage TLR4 and the receptor for advanced glycation end products (RAGE) on neutrophils, thereby promoting PAD4-dependent histone citrullination and NET formation [52,53]. Platelet–neutrophil interactions within the tumor vasculature provide an additional amplification loop. Activated platelets release thromboxane A2 (TXA2) and platelet-activating factor (PAF), which prime neutrophils for NETosis [54], whereas activation of platelet TLR4 by LPS or related microbial signals can further promote rapid NET formation [47]. Cancer stem cells (CSCs) and CSC-enriched tumor subpopulations also secrete HMGB1, interleukin-8 (IL-8), and CXCL5, thereby recruiting neutrophils and promoting their acquisition of a NET-forming phenotype [55]. In turn, NET-derived NE and extracellular DNA can induce epithelial–mesenchymal transition (EMT) in tumor cells, establishing a reciprocal feed-forward loop that reinforces tumor progression [56]. Collectively, these tumor-specific mechanisms support the development of context-selective strategies that disrupt pathological NET formation while preserving systemic neutrophil function.

### 2.4. Mitochondrial DNA (mtDNA)-Derived NETs (mtNETs)

A mechanistically distinct form of NET formation involves the release of mitochondrial rather than nuclear DNA. Mitochondrial DNA-containing NETs (mtNETs) have been reported to contain oxidized mitochondrial DNA (ox-mtDNA), which may exhibit heightened immunogenicity owing to its hypomethylated, CpG-rich composition and structural resemblance to bacterial DNA [57]. In some forms of vital NETosis, neutrophils can extrude mtDNA without undergoing immediate cell death through a process involving small-conductance calcium-activated potassium channel 3 (SK3)-dependent Ca^2+^ signaling and mitochondrial reactive oxygen species (mtROS) production. This pathway appears to proceed independently of NOX activity, although PAD4 may still contribute to NET formation [58]. Within the TME, ox-mtDNA released during mtNET formation has been proposed to activate the cyclic GMP–AMP synthase–stimulator of interferon genes (cGAS–STING) pathway, potentially eliciting type I interferon responses with context-dependent consequences, ranging from enhanced antitumor immunity to the induction of interferon-driven immunosuppressive programs [57]. Although direct evidence for this mechanism in tumor-associated NETs remains limited, extracellular mtDNA released by senescent tumor cells has been shown to activate cGAS-STING-NF-κB signaling in polymorphonuclear myeloid-derived suppressor cells (PMN-MDSCs), thereby enhancing immunosuppressive activity in the TME [59]. The distinct molecular composition and immunological properties of mtNETs highlight the value of function-based NET classification for the development of precision therapeutic strategies.

### 2.5. PAD Enzyme Isoforms in Tumor-Associated NETosis

Although PAD4 is the predominant PAD isoform responsible for histone citrullination and NET formation in neutrophils, accumulating evidence indicates that PAD2 also contributes to tumor-associated NETosis and broader remodeling of the TME. The biological effects of PAD2 appear to be highly dependent on tumor type, disease stage and cellular compartment, rather than being uniformly pro- or antitumorigenic [60,61]. PAD2 is expressed in tumor-associated neutrophils and macrophages, where it may mediate histone citrullination during NETosis as well as the modification of non-histone substrates. For example, PAD2-mediated citrullination of the vimentin has been associated with its extracellular release and may contribute to remodeling of the ECM [62]. Proteomic analyses further suggest that ECM citrullination within tumors is predominantly attributable to PAD activity from infiltrating inflammatory cells rather than cancer cells or cancer-associated fibroblasts (CAFs) [63].

Importantly, studies in colorectal cancer (CRC) and pancreatic ductal adenocarcinoma (PDAC) illustrates the context-dependent roles of PAD2 in cancer. In CRC, PAD4 has been implicated in ECM citrullination during liver metastasis, whereas PAD2 may exert tumor-suppressive effects in primary tumors [64]. In contrast, PAD2 has been associated with tumor-promoting transcriptional and macrophage-related programs in PDAC [65]. These findings indicate that PAD2 can influence tumor biology through distinct mechanisms from PAD4-mediated NETosis.

This distinction has important implications for interpreting PAD-targeted NETosis therapies. Pan-PAD inhibitors may suppress PAD4-dependent NET formation while simultaneously altering PAD2-mediated functions that may be either tumor-suppressive or tumor-promoting depending on the disease context [64,65]. Consequently, the anti-tumor effects of PAD inhibition should not be attributed solely to NET inhibition. Future studies should delineate PAD4-dependent NETosis from PAD2-mediated tumor and stromal effects and determine whether isoform-selective PAD4 inhibition can effectively suppress NET formation while preserving potentially beneficial PAD2 functions.

### 2.6. Gasdermin D (GSDMD)-Dependent NETosis: Convergence of Canonical and Non-Canonical Inflammasome Pathways

Beyond the non-canonical caspase-4/5/11 pathway described above, GSDMD-dependent NET release has also been reported downstream of canonical NLRP3 and AIM2 inflammasome activation. Within the TME, tumor-derived danger signals, including uric acid [66], extracellular ATP [67], and cytosolic double-stranded DNA (dsDNA) sensed by AIM2 [68,69], may activate these inflammasomes, leading to the recruitment and activation of caspase-1. Cleavage of GSDMD by caspase-1 in canonical inflammasome pathways, or by caspase-4/5/11 in non-canonical pathways, releases its pore-forming N-terminal fragment. This fragment forms membrane pores that promote ionic fluxes, cellular swelling, and the extrusion of NET-like chromatin structures [41,70,71].

Because these convergent pathways can proceed independently of NOX activity and may not invariably require PAD4 activation, GSDMD-dependent NETosis initiated through either canonical or non-canonical inflammasome signaling may be less susceptible to conventional NETosis inhibitors that target ROS production or PAD4 activity [41]. NETs generated through these pathways may retain or become associated with pro-inflammatory cytokines, including IL-1β and IL-18, thereby amplifying local inflammatory signaling and potentially contributing to immunoregulatory or immunosuppressive features of the TME. Recognition of this mechanistic convergence supports the development of combinatorial strategies targeting both PAD4-dependent and GSDMD-dependent NETosis in cancer.

## 3. Roles of NETosis in the Tumor Immune Microenvironment

### 3.1. Regulation of the Tumor Microenvironment (TME)

NETs are abundantly induced across a wide spectrum of malignancies, including hepatocellular, colorectal, pancreatic, breast, gastric, lung, and hematological cancers, where they remodel the TME through multiple mechanisms. NET-associated proteases, ox-mtDNA, and inflammatory mediators have been implicated in promoting tumor-supportive inflammation, angiogenesis, ECM remodeling, and EMT, thereby establishing a permissive microenvironment that facilitates tumor survival, invasion, and metastatic dissemination [72,73]. Notably, TME architecture differs fundamentally between solid and hematological malignancies. Solid tumors exhibit spatially organized tumor-stroma interactions, where hematological malignancies arise predominantly within specialized bone marrow or lymphoid niches characterized by distinct stromal, vascular, and immune-cell organization [74]. These architectural differences may shape the relative contribution of NET-mediated physical scaffolding and molecular immunomodulation across tumor types and should be considered when translating NET-targeted strategies between solid and hematological malignancies.

### 3.2. Effects on Immune Cells

**Figure 3:**
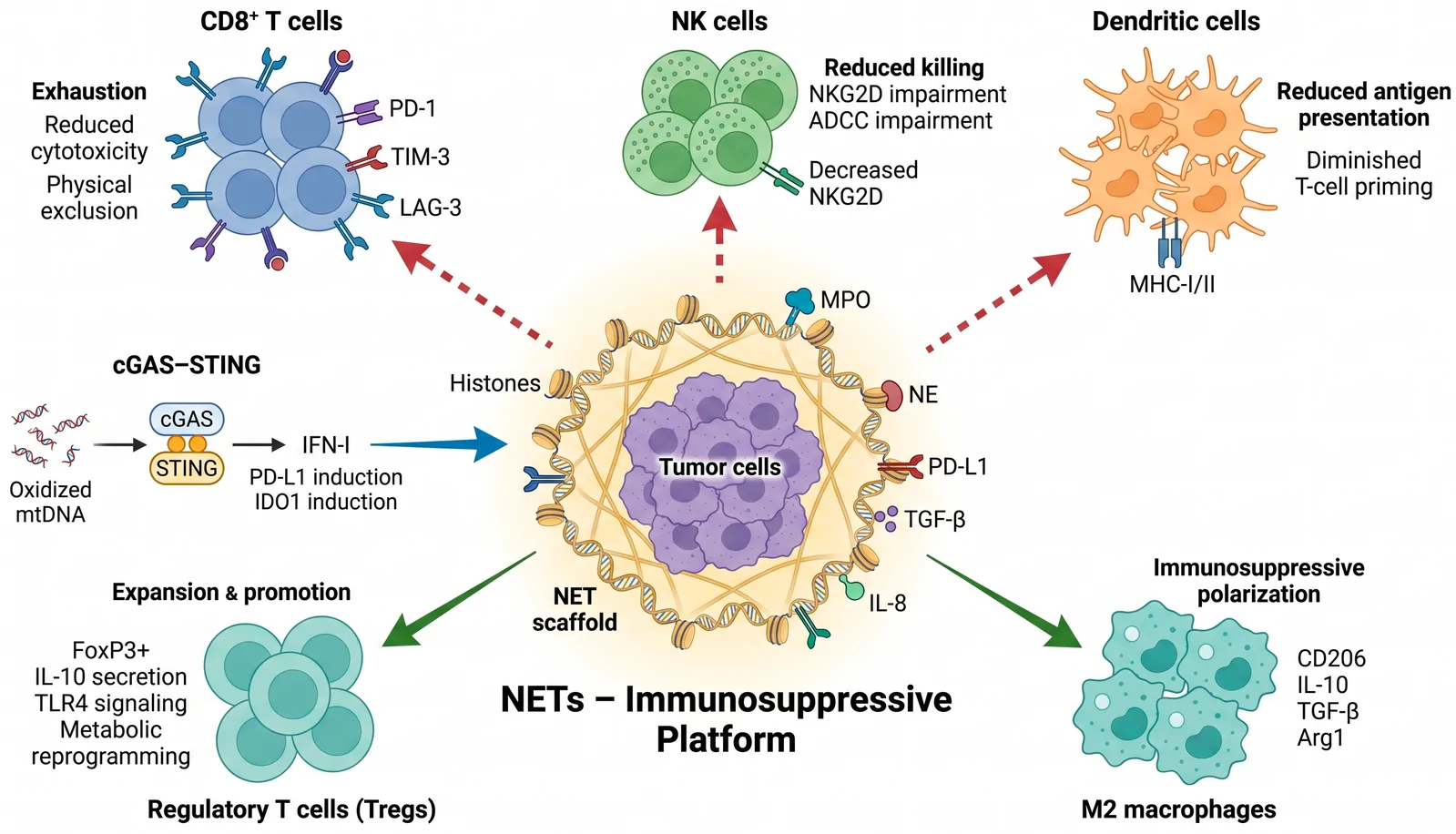
Multifaceted immunomodulatory roles of NETs in the TME. Neutrophil extracellular traps (NETs) form a physical and molecular scaffold around tumor cells and carry immunoregulatory components, including programmed death-ligand 1 (PD-L1), transforming growth factor-β (TGF-β), interleukin-8 (IL-8), myeloperoxidase (MPO), neutrophil elastase (NE), and histones. NETs promote CD8^+^ T-cell exhaustion and exclusion, impair natural killer (NK)-cell cytotoxicity, reduce dendritic-cell antigen presentation, expand regulatory T cells, and drive immunosuppressive macrophage polarization. Oxidized mitochondrial DNA (mtDNA) may further activate cyclic GMP–AMP synthase–stimulator of interferon genes (cGAS–STING) signaling, inducing type I interferon, PD-L1, and indoleamine 2,3-dioxygenase 1 (IDO1) expression. Collectively, these effects suppress antitumor immunity and facilitate immune evasion. Figure is created in BioRender. Su, Y. (2026) https://BioRender.com/sri33ej.

NETs modulate both innate and adaptive immune compartments, thereby reshaping the tumor immune microenvironment (Figure 3). They can induce apoptosis or promote functional reprogramming of macrophages toward an M2-like phenotype and dendritic cells toward a tolerogenic state, while altering cytokine production and impairing antigen presentation [13].

NET-associated components, including cathepsin G and histones, have been implicated in suppressing natural killer (NK) cell activation and cytotoxicity. NETs may also form physical barriers that restrict NK-cell infiltration and promote NK-cell dysfunction through mechanisms involving angiopoietin-2 and inflammatory cytokines, including IL-2 and IL-15 [75]. In addition, NET-bound histones have been reported to promote T helper 17 (Th17) cell differentiation through a TLR2–MyD88–STAT3-dependent signaling pathway [70]. NETs can also drive the differentiation of naive CD4^+^ T cells toward a regulatory T-cell (Treg) phenotype by inducing TLR4-dependent metabolic reprogramming toward oxidative phosphorylation, a mechanism implicated in the progression from non-alcoholic steatohepatitis to hepatocellular carcinoma (HCC) [76].

In parallel, NET accumulation has been associated with reduced CD8^+^ T-cell infiltration and increased expression of exhaustion-associated markers, including PD-1, TIM-3, and LAG-3 [13,46]. NETs may also modulate B-cell activation and differentiation, thereby contributing to humoral immune dysregulation within the tumor-infiltrating B-cell compartment [77]. Moreover, NETs can function as immunosuppressive scaffolds that concentrate immunoregulatory mediators, including PD-L1, transforming growth factor-β (TGF-β), and IL-8, while promoting the recruitment and activation of PMN-MDSCs. Together, these effects may reinforce immune exclusion within the TME.

### 3.3. Association with Tumor Progression

Experimental and clinical evidence indicates that NETs promote primary tumor progression by inducing EMT, stem-like phenotypes, and invasive behavior through multiple signaling pathways, including TLR4–p38 MAPK–peroxisome proliferator-activated receptor-γ coactivator 1α (PGC-1α) [78], IL-1β–epidermal growth factor receptor (EGFR)–ERK, TLR9–nuclear factor-κB (NF-κB)–signal transducer and activator of transcription 3 (STAT3)–p38 MAPK, and HMGB1–RAGE–IL-8 signaling [27]. During metastatic dissemination, NETs facilitate tumor cell detachment, migration, and invasion; capture circulating tumor cells through β1-integrin-mediated interactions [79,80]; protect them from hemodynamic shear stress and immune-mediated elimination; and promote colonization of distant organs [46,73]. In addition, NETs have been implicated in the reactivation of dormant tumor cells and in protecting cancer cells from chemotherapy- and radiotherapy-induced cytotoxicity, thereby promoting immune evasion and therapeutic resistance [72].

At the structural level, NETs may function not only as a source of signaling mediators but also as a within the TME. Decondensed chromatin fibers and NET-associated histones from extracellular meshworks that can influence immune-cell trafficking and interactions with the surrounding ECM. NET-associated proteases, including NE, MMP-9, and cathepsin G, can further modify ECM components and tumor-associated signaling molecules, thereby promoting tissue remodeling and tumor invasion [27,72]. In desmoplastic tumors such as PDAC, CAF-derived IL-8 can promote neutrophil recruitment and NET formation through CXCR1/2 signaling, suggesting a feed-forward interaction between stromal inflammation and NET accumulation [81]. Collectively, these findings support a model in which NETs contribute to tumor progression and immune exclusion through the coordinated effects of molecular signaling and physical remodeling of the TME.

### 3.4. Cancer-Associated Fibroblasts (CAFs) and NETs: A Pro-Tumorigenic Feedback Loop

CAFs represent a key but often underappreciated component of NET-driven remodeling of the TME. CAF-derived cytokines, including interleukin-6 (IL-6) and IL-8, create a chemotactic and activating milieu for tumor-infiltrating neutrophils. In pancreatic cancer specimens, neutrophils co-localize with IL-6-positive CAFs, whereas exposure to CAF-conditioned medium enhances neutrophil migration and prolongs neutrophil survival. These changes promote the acquisition of pro-tumorigenic neutrophil functions that can be attenuated pharmacologically by the antifibrotic agent pirfenidone [82]. Independently, NET formation has been linked to a prothrombotic, tumor-supportive state. Tumor-bearing mice exhibit increased NET formation and a hypercoagulable phenotype even in the absence of additional inflammatory stimuli, providing early experimental evidence of a direct association between malignancy and NET generation [83]. This CAF–neutrophil crosstalk is particularly well characterized in PDAC, which is defined by a dense desmoplastic stroma that restricts drug delivery and immune-cell infiltration. However, the contribution of NET-induced fibroblast activation to this desmoplastic phenotype, as distinct from CAF-mediated neutrophil activation, has not been directly established and remains an important area for further investigation.

### 3.5. NETs, B Cells and Tertiary Lymphoid Structures

The interplay between NETs and B-cell-mediated immunity has been most clearly established in autoimmune diseases, where NETs provide a sustained source of citrullinated autoantigens, particularly citrullinated histones, that are recognized by autoreactive B cells generated within ectopic lymphoid structures. This process drives the production of anti-citrullinated protein antibodies (ACPAs) in rheumatoid arthritis [84] and the formation of interferogenic immune complexes in systemic lupus erythematosus [57]. Whether an analogous NET-driven B-cell activation loop operates within the TME remains largely unexplored. In tumors, NET-rich regions are associated with reduced infiltration and impaired function of CD8^+^ T cells and NK cells [46], whereas tertiary lymphoid structures (TLSs), organized aggregates of B and T cells, are consistently associated with improved responses to ICIs in melanoma [85,86]. Whether NET-mediated immunosuppression directly impairs TLS neogenesis or disrupts the humoral immune functions supported by TLSs has not yet been determined and represents an important area for future investigation.

### 3.6. Cancer-Specific NET Profiles

The functional consequences of NETosis vary across tumor types (Table 1), reflecting differences in NET-inducing signals, stromal architecture, and immune contexture [27]. In HCC, NET formation is increased in patients with portal vein tumor thrombosis (PVTT) and is associated with poor clinical outcomes. Mechanistically, crosstalk between tumor cells and neutrophils promotes HCC invasion and metastasis through NET-associated cathepsin G activity [87]. In addition, a non-invasive computed tomography (CT) radiomics-based NET signature, developed and validated in 1133 patients across five independent HCC cohorts, has been shown to predict clinical outcomes and response to immunotherapy [88].

In PDAC, gemcitabine induces tumor cells to secrete IL-8, which activates CXCR1/2 on neutrophils and promotes chemotherapy-induced NET formation, termed chemoNETosis. Pharmacological blockade of CXCR1/2 with navarixin suppresses chemoNETosis, restores gemcitabine sensitivity, and significantly reduces tumor growth in vivo, thereby identifying NETs as mediators of chemoresistance in PDAC [81]. In NSCLC, tumor-derived CXCL5 recruit neutrophils and induces PD-L1 upregulation through paxillin (PXN)–protein kinase B (AKT) signaling, forming a positive feedback loop. These PD-L1^+^ neutrophils suppress CD8^+^ T-cell-dependent antitumor immunity, whereas elevated CXCL5 expression is associated with poor clinical outcomes [89].

In CRC, NETs generated following hepatectomy in a model of CRC liver metastasis promote T-cell exhaustion and dysfunction. Degradation of NETs with deoxyribonuclease I (DNase I) restores CD8^+^ T-cell function and significantly suppresses tumor growth [13].

In breast cancer, TRAPs induce PD-L1-decorated NETs through the HMGB1–TLR4–MyD88–ERK/p38 MAPK signaling axis [14]. These PD-L1-decorated NETs suppress T-cell function and promote pulmonary pre-metastatic niche formation. Notably, combined treatment with DNase I and PD-L1 blockade reverses these effects without compromising antitumor efficacy.

### 3.7. NETs and Cancer Stem Cell (CSC) Maintenance

Emerging evidence supports a bidirectional relationship between NET formation and CSC biology. Exposure to NETs promotes EMT in tumor cells, as evidenced by upregulation of the transcription factors Snail family transcriptional repressor 1 (SNAI1), Snail family transcriptional repressor 2 (SNAI2), and zinc finger E-box-binding homeobox 1 (ZEB1); loss of E-cadherin; and increased expression of N-cadherin, vimentin, fibronectin, and β-catenin. This NET-induced EMT program is accompanied by acquisition of a CD44^high^/CD24^low^ stem-like phenotype in breast cancer cells [56]. Because CSCs are intrinsically more resistant to cytotoxic and immune-mediated elimination than bulk tumor cells, NET-driven expansion of this population may contribute to therapeutic resistance and disease recurrence. Conversely, CSCs secrete elevated levels of HMGB1, IL-8 and CXCL5, thereby establishing chemotactic gradients that recruit and activate neutrophils and promote further NET formation [55]. Together, these reciprocal interactions create a self-reinforcing loop between NET formation and tumor cell stemness, representing a rational, although still predominantly preclinical, target for combination therapeutic strategies.

## 4. Relationship Between NETosis and Immune Checkpoint Inhibitor (ICI) Efficacy

### 4.1. Impact of NETs on Checkpoint Blockade

**Figure 4:**
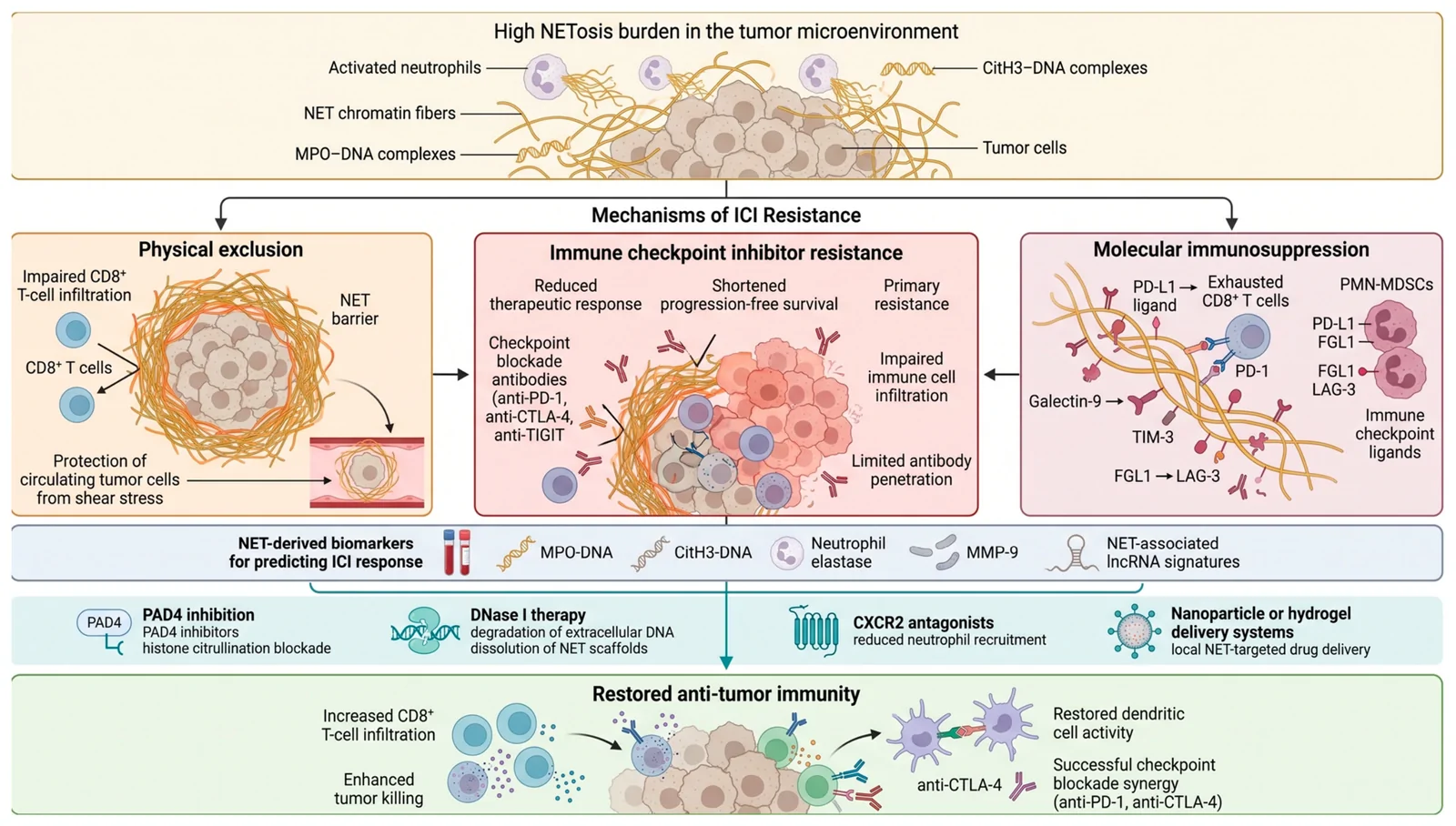
NET-mediated mechanisms of immune checkpoint inhibitor resistance and therapeutic opportunities. High neutrophil extracellular trap (NET) burden within the TME promotes resistance to immune checkpoint inhibitors (ICIs) through complementary physical and molecular mechanisms. NET scaffolds physically exclude cytotoxic lymphocytes and protect circulating tumor cells, whereas NET-associated immunoregulatory molecules, including programmed death-ligand 1 (PD-L1), transforming growth factor-β (TGF-β), myeloperoxidase (MPO), neutrophil elastase (NE), histones, and peptidyl arginine deiminase 4 (PAD4)-dependent chromatin components, promote CD8^+^ T-cell exhaustion, polymorphonuclear myeloid-derived suppressor cell (PMN-MDSC)-mediated immunosuppression, and immune checkpoint inhibitor resistance. Circulating and tissue-derived NET biomarkers, including MPO–DNA, citrullinated histone H3 (CitH3)–DNA, NE, matrix metalloproteinase-9 (MMP-9), and NET-associated long non-coding RNA (lncRNA) signatures, may facilitate prediction of ICI response. Therapeutic strategies targeting NETs—including PAD4 inhibition, deoxyribonuclease I (DNase I)-mediated NET degradation, C-X-C chemokine receptor 2 (CXCR2) blockade, and tumor-targeted NET-directed delivery systems—may restore immune-cell infiltration, enhance antitumor immunity, and improve responses to ICIs and cell-based immunotherapies. Figure is created in BioRender. Su, Y. (2026) chttps://BioRender.com/4uae1kh.

Structurally, NETs can physically entrap tumor cells and restrict their direct interactions with cytotoxic CD8^+^ T cells and NK cells, thereby reducing the efficacy of PD-1/PD-L1 and cytotoxic T-lymphocyte-associated protein 4 (CTLA-4) blockade [13,46]. Given this shared physical-barrier mechanism, NET-mediated impairment of chimeric antigen receptor T-cell (CAR-T-cell) engagement is mechanistically plausible, although it has not yet been directly demonstrated. At the molecular level, NETs function as immunosuppressive platforms enriched in PD-L1 and other mediators that promote T-cell exhaustion (see Section 3.2 for detailed mechanisms; Figure 4). Together, these structural and molecular effects create a barrier to effective antitumor immunity within NET-rich TMEs.

### 4.2. Evidence from Clinical and Preclinical Studies

Preclinical models of PDAC demonstrate that high intratumoral NET density is associated with poor responses to anti-PD-1 therapy, whereas NET degradation or inhibition sensitizes tumors to ICI by enhancing CD8^+^ T-cell infiltration and effector function [90]. Consistent with these findings, NETs generated following hepatectomy in a model of CRC liver metastasis promote T-cell exhaustion and dysfunction, whereas DNase I-mediated NET degradation restores CD8^+^ T-cell function and suppresses tumor growth [13]. In bladder cancer models, radiotherapy-induced NETs contribute to radioresistance, and inhibition of NET formation using NE inhibitors or DNase I enhances tumor responses to radiotherapy [91], supporting a broader role for NETosis in resistance to radiotherapy and systemic anticancer therapies across multiple tumor types [92]. Mechanistically, primary tumors can induce PD-L1-enriched NETs that suppress T-cell function [13,46], consistent with the immunosuppressive mechanisms described in Section 3.2. These findings are corroborated by studies demonstrating that PAD4 inhibition or deficiency enhances ICI responses in murine models [49,72], as discussed in detail in Section 5.2.

### 4.3. NETosis-Related Biomarkers

Circulating and tissue-based markers of NETs have been associated with tumor burden and adverse clinical outcomes in a tumor-type-specific manner. In HCC, NETs are enriched in ox-mtDNA, which is associated with pro-inflammatory and pro-metastatic phenotypes [93]. In a retrospective cohort of patients with bladder cancer, elevated serum myeloperoxidase–DNA (MPO–DNA) complexes and tumor CitH3 positivity were independently associated with reduced benefit from ICIs, metastatic disease, and shorter PFS, supporting their potential utility as predictive biomarkers [94]. Separately, circulating CitH3 has been validated as a prognostic biomarker associated with an increased risk of mortality in patients with cancer [25]. Whether these NET-associated markers can also predict susceptibility to immune-related adverse events (irAEs) remains unknown and warrants prospective evaluation.

### 4.4. Next-Generation Checkpoints and NETs: TIGIT, LAG-3 and TIM-3

Beyond the PD-1/PD-L1 axis, exposure to NETs promotes T-cell exhaustion, characterized by upregulation of multiple co-inhibitory receptors; reduced production of effector cytokines, including IL-2, interferon-γ (IFN-γ), and tumor necrosis factor-α (TNF-α); and metabolic dysfunction, including impaired mitochondrial activity and glucose uptake [13]. This phenotype is consistent with the co-expression of PD-1, TIM-3, and LAG-3, which is frequently observed in terminally dysfunctional CD8^+^ tumor-infiltrating lymphocytes in melanoma and other solid tumors [16]. Several additional immunosuppressive pathways are independently established within the TME, including galectin-9–TIM-3 signaling [16]; FGL1, a major inhibitory ligand of LAG-3 [17]; and the poliovirus receptor (PVR, also known as CD155)/CD112–TIGIT axis. The latter suppresses natural killer (NK)-cell cytotoxicity and can be counteracted by combined IL-15 stimulation and TIGIT blockade [18]. Because tumor-associated neutrophils and PMN-MDSCs are enriched in NET-rich microenvironments, these pathways may converge with NET-mediated immunosuppression. However, whether NETs directly display ligands for TIM-3, LAG-3, or TIGIT, analogous to their established capacity to carry PD-L1, remains unknown. This question is particularly relevant given the growing clinical interest in dual- and triple-checkpoint blockade strategies.

### 4.5. NET Biomarkers and Combination Strategies for ICI Therapy

Circulating NET-derived markers are emerging as promising tools for stratifying patients receiving ICI therapy. In NSCLC, a composite model incorporating serum NET markers, CD8^+^ T-cell infiltration, and PD-L1 tumor proportion score (TPS) improved prediction of responses to ICIs [95]. In addition, a NET-associated long non-coding RNA (lncRNA) signature independently stratified prognosis and immune checkpoint gene expression in lung adenocarcinoma [96].

Beyond treatment efficacy, single-cell transcriptomic analysis of patients who developed irAEs revealed increased expression of NET-related genes, including PADI4, cytochrome b-245 beta chain (CYBB), neutrophil cytosolic factor 1 (NCF1), and neutrophil cytosolic factor 2 (NCF2), in circulating neutrophils [94]. In a murine model, combining NET degradation with PD-1 blockade attenuated treatment-associated hepatic and renal inflammatory injury and reduced circulating levels of IL-6, TNF-α, and TGF-β, without compromising antitumor efficacy [94]. Together, these findings suggest that composite NET biomarker panels may have the potential to predict both treatment response and immune-related toxicity, although prospective validation across additional tumor types is required.

Therapeutically, the combination of NET-targeting agents (PAD4 inhibitors, DNase I) with ICI blockade has demonstrated synergistic anti-tumor efficacy across multiple murine models [49,97], with detailed mechanisms and preclinical evidence presented in Section 5.2. Notably, combined NET degradation with PD-1 blockade simultaneously enhances antitumor efficacy and attenuates immune-related toxicity in a murine bladder cancer model [94].

Clinical translation of these strategies remains at an early stage, and no dedicated clinical trials combining NET-targeting agents with ICIs in solid tumors have yet been reported. Prospective, biomarker-stratified trials incorporating longitudinal and paired measurements of tissue and circulating NET markers will be essential to establish safety and efficacy, identify patients most likely to benefit, and define clinically actionable frameworks for integrating NET modulation with immunotherapy.

### 4.6. NETs as Barriers to CAR-T and NK Cell Therapies in Solid Tumors

The physical and molecular barriers imposed by NETs may be particularly relevant to cell-based immunotherapies targeting solid tumors. Within the tumor stroma, the chromatin-rich NET scaffold has been proposed to act as a spatial barrier that limits direct interactions between CAR-T cells and tumor cells, potentially contributing to the lower efficacy of CAR-T-cell therapy in solid tumors than in hematological malignancies [98]. Degradation of NETs with DNase I therefore represents a mechanistically rational strategy to facilitate CAR-T-cell access to tumor tissue. However, this effect has not yet been directly or systematically quantified in dedicated preclinical models.

For NK-cell-based therapies, NETs impair antitumor NK-cell function through multiple mechanisms, including the promotion of platelet-derived TGF-β release, which establishes an immunosuppressive niche that suppresses NK-cell cytotoxic activity [99]. Given the established role of NET-PD-L1 in T-cell exhaustion (Section 3.2) [13], a mechanistically analogous inhibitory effect on NK cells is plausible, although it remains to be directly demonstrated. Collectively, these observations support NET-disrupting strategies as a rational but still preclinically underdeveloped approach to improving cell therapy delivery and efficacy in immunologically cold solid tumors.

## 5. Therapeutic Strategies Targeting NETosis

### 5.1. Pharmacologic Inhibition of NET Formation or Persistence

Several therapeutic strategies can inhibit NETosis or dismantle pre-existing NETs. PAD4 inhibitors, including GSK484 and BMS-P5, suppress histone citrullination and NET formation, thereby limiting tumor growth, metastatic dissemination, and reactivation of dormant cancer cells in preclinical models [100]. Direct enzymatic degradation of NETs with DNase I reduces the physical trapping of tumor cells and improves chemotherapeutic drug penetration within the TME [101]. Targeting upstream inflammatory signals and neutrophil-recruiting pathways may likewise prevent tumor-induced NET formation. For example, inhibition of cathepsin C disrupts the IL-1β–p38 MAPK–ROS signaling axis that promotes NETosis during breast cancer metastasis [102]. Antagonism of CXCR1/2 inhibits tumor-derived chemokine-driven NET formation [46], whereas blockade of complement component 5a (C5a) and its receptor C5a receptor 1 (C5aR1) reduces PMN-MDSC-mediated NETosis and circulating tumor cell burden [99]. Inhibition of platelet TLR4, through which platelets sense LPS and promote NET release, may further limit platelet-primed NETosis within the tumor vasculature [47]. JBI-589 and other next-generation PAD4 inhibitors additionally downregulate CXCR2, restrict neutrophil and PMN-MDSC trafficking, and enhance the antitumor activity of PD-1/PD-L1 blockade in preclinical models [49].

### 5.2. Combination of NETosis Modulation with Immunotherapy

Evidence from murine models indicates that combining NET inhibition, through DNase I treatment or PAD4 blockade, with PD-1 blockade or dual PD-1/CTLA-4 blockade improves tumor control by increasing cytotoxic T-cell infiltration, reversing T-cell exhaustion, and disrupting NET-mediated immune exclusion [13,49]. Given the physical barrier functions of NETs described in Section 3.3 and Section 4.1, NET-targeting strategies may also enhance NK cell-based and CAR-T-cell therapies in solid tumors (Section 4.6). In addition, disruption of NETs could facilitate CAR-T-cell trafficking and tumor-cell engagement in solid tumors, although this possibility remains to be directly validated [46].

NET-loaded dendritic cell vaccines have also shown proof-of-concept activity in nucleophosmin 1 (NPM1)-mutant myeloproliferative neoplasm models, suggesting that controlled exploitation of NET-associated antigens may be harnessed for therapeutic cancer vaccination [103]. Beyond NET-based vaccine strategies, the role of NETs in conventional antitumor vaccine platforms remains poorly understood. Notably, a recent preclinical study demonstrated that lipid nanoparticles used for mRNA vaccine delivery can induce neutrophil activation and NETosis through mtDNA-associated mechanisms, suggesting that a potential interplay between vaccine delivery systems and NET biology in shaping antitumor immunity [104]. However, whether NET formation enhances or impairs the therapeutic efficacy of mRNA-, DNA-, or peptide-based cancer vaccines remains largely unexplored.

In parallel, multiple NET-targeting strategies, including DNase-mediated NET degradation, CXCR1/2 antagonism, and TGF-β blockade, have demonstrated efficacy in reversing immune exclusion across preclinical tumor models. Building on these findings, CXCR1/2 antagonists are beginning to enter early-phase clinical evaluation in combination with immune checkpoint blockade, supporting the translational potential of NET-directed therapeutic strategies [105].

Novel nanocarrier and hydrogel platforms further enable the localized and sustained delivery of NET-degrading enzymes. For example, a dual pH-responsive injectable hydrogel that co-delivers a tumor acidity-neutralizing agent and DNase I enhanced adoptive NK-cell therapy and prevented post-resection recurrence in HCC models [106]. Similarly, NET-targeting, DNase I-loaded, cell membrane-derived liposomes suppressed CRC liver metastasis in mice [107].

### 5.3. Ongoing and Preclinical Development

Current efforts remain largely preclinical, with studies exploring PAD4 inhibitors [108], DNase delivery systems, CXCR2 antagonists, complement-targeting agents, and biomaterial-based NET-lytic platforms in combination with ICIs or adoptive cell therapies [109]. Collectively, these studies support the integration of NET-targeting approaches into rational combination regimens designed to overcome immunotherapy resistance and improve durable tumor control (Table 2).

### 5.4. Clinical Development Status of PAD4 Inhibitors

Several PAD4 inhibitors remain in preclinical development, each with distinct pharmacological properties. GSK484 is a reversible, competitive inhibitor with high selectivity for PAD4 over PAD1-3 and favorable pharmacokinetic properties in rodent models, including a good volume of distribution and a suitable half-life; it has demonstrated efficacy in preventing inflammation-induced awakening of dormant cancer cells in murine breast cancer models [72]. However, its relatively modest cellular potency, requiring micromolar concentrations to achieve effective PAD4 inhibition in vitro, may limit clinical translation [111]. BMS-P5 is a more potent PAD4-selective inhibitor that suppresses multiple myeloma-induced NET formation and delays disease progression in syngeneic mouse models [111]. Unlike the covalent, non-selective pan-PAD inhibitors Cl-amidine and BB-Cl-amidine, BMS-P5 is mechanistically and structurally distinct, providing greater PAD4 selectivity and an improved off-target profile. JBI-589 is an orally bioavailable PAD4-selective inhibitor that downregulates CXCR2 expression, reduces PMN-MDSC infiltration, and enhances responses to PD-1 and CTLA-4 blockade in murine tumor models [49]. Collectively, these agents support the therapeutic potential of selective PAD4 inhibition, although no PAD4 inhibitor has yet entered clinical trials for oncology indications.

Future priorities include establishing first-in-human safety and tolerability, particularly with respect to infection risk given the role of PAD4 in innate host defense, optimizing oral bioavailability and tumor-selective drug delivery, and developing biomarker-guided patient selection strategies based on baseline PAD4 expression and circulating NET markers [130]. The context-dependent roles of PAD2 and PAD4 discussed above (Section 2.5) should be carefully considered when translating PAD4 inhibition into clinical application. Selective inhibition of PAD4 may provide a more precise strategy for suppressing NET formation while preserving PAD2-mediated, NET-independent functions. However, the potential consequences of PAD4 inhibition for host antimicrobial defense warrant careful evaluation during clinical development.

### 5.5. Nanoparticle and Biomaterial Platforms for Localized NET Modulation

Systemic administration of NET-disrupting agents carries inherent risks of impairing antimicrobial host defense, given the established role of PAD4 and NETs in antibacterial innate immunity [36]. This concern has motivated the development of tumor-targeted delivery platforms. Tumor-homing and tumor-penetrating peptide technologies, such as the internalizing arginine–glycine–aspartic acid (iRGD) platform, provide an established approach for promoting selective intratumoral drug accumulation and could potentially be adapted for the delivery of DNase I [131]. Hydrogel-based platforms co-delivering a tumor acidity-neutralizing agent and DNase I have enhanced the efficacy of adoptive NK-cell therapy and reduced NET burden in a murine HCC resection model [106]. More recently, DNase I-conjugated cerium oxide nanoparticles have been engineered to combine intrinsic ROS-scavenging activity with enzymatic NET degradation. This dual-functional platform prevented NET formation and dismantled pre-existing NET structures in an in vitro model of immunothrombosis [132]. Whether this approach can be adapted to suppress tumor-associated NETs and improve immune-cell trafficking, including T-cell motility, remains to be determined in relevant tumor models.

### 5.6. Balancing NET Suppression with Host Defense

A central safety concern in NET-targeted oncology is the potential impairment of innate host defense against bacterial and fungal infections, given the established role of NETs in pathogen capture and killing. In a murine model of necrotizing fasciitis, global PAD4 deficiency increased susceptibility to group A Streptococcus infection, underscoring this potential risk [36]. Nevertheless, several lines of evidence suggest that NET modulation may be achievable within a clinically acceptable therapeutic window. First, tumor-selective delivery platforms, including nanoparticles and hydrogels, are designed to minimize systemic neutrophil impairment. However, direct evidence that circulating neutrophils retain normal ex vivo NET-forming capacity following such treatment remains lacking. Second, circulating CitH3–DNA complexes are measurable markers of NET burden, raising the possibility that partial, rather than complete, PAD4 inhibition could be guided by pharmacodynamic monitoring to reduce pathological NET formation while preserving antimicrobial function [25]. This strategy, however, has not yet been evaluated in infection-challenge models. Third, the timing of NET-targeted treatment relative to chemotherapy-induced neutropenia requires careful consideration, because NET suppression during periods of profound neutropenia could further increase infection risk. Future clinical trials of PAD4 inhibitors or DNase-based agents should therefore incorporate systematic infection surveillance, risk-adapted antimicrobial prophylaxis, and predefined dose-modification criteria informed by pharmacodynamic NET markers, neutrophil counts, and clinical evidence of infection. Defining the therapeutic window for NET modulation remains a major translational challenge in oncology.

## 6. Research Challenges and Future Directions

### 6.1. Limitations of Current Evidence

Key limitations include the lack of standardized quantitative assays for NETs, the inability to distinguish lytic from vital NETosis in clinical specimens, and unresolved compositional heterogeneity among NETs, including PD-L1-high versus PD-L1-low NETs [13] and mtDNA-enriched NET subsets [93]. This heterogeneity may have direct clinical relevance, as NET-low tumor-associated neutrophil phenotype has been associated with improved PFS and OS following first-line immunotherapy in patients with advanced NSCLC [133].

Despite growing interest in NETs as clinical biomarkers, available clinical studies remain limited and heterogeneous in tumor type, NET markers, treatment context and clinical endpoints (Table 3). Most studies are observational and primarily evaluate circulating or tissue-associated NET markers, whereas prospective interventional studies directly targeting NETs remain scarce. These limitations underscore the need for standardized NET assessment and prospective validation of NET-based biomarkers in oncology.

### 6.2. Unsolved Questions

Key unresolved issues include the context-dependent balance between the pro- and antitumor effects of NETs, the relative contributions of distinct NET components to immune modulation, and the crosstalk among NETs, MDSCs, and stromal elements within the TME. It also remains unclear which patient populations are most likely to benefit from NET-targeted therapies, how these interventions should be optimally integrated with ICIs or chemotherapy, and how impairment of antimicrobial host defense can be minimized. Because systemic NET inhibition may compromise innate immunity and increase susceptibility to infection, selective modulation of tumor-associated NETs will be critical for achieving an acceptable therapeutic window and ensuring the safe clinical development of NET-targeted therapies.

A particularly important unresolved question is the bidirectional crosstalk between CAFs and NETs within the desmoplastic TME. Although CAF-derived cytokines, including IL-6 and IL-8, have been implicated in neutrophil recruitment and NET formation in pancreatic cancer [82], whether NETs or NET-associated components directly promote CAF activation and extracellular matrix remodeling remains poorly understood. Advanced three-dimensional organoid and tumor-on-chip co-culture systems incorporating tumor cells, CAFs, and neutrophils, combined with intravital imaging and single-cell or spatial profiling, may help distinguish direct NET-CAF interactions from secondary inflammatory effects. These approaches could clarify whether CAFs and NETs form a self-reinforcing feedback loop and define the therapeutic relevance of disrupting this axis in desmoplastic tumors.

### 6.3. Future Technologies and Directions

Recent advances in single-cell, spatial transcriptomic and multi-omics technologies have begun to resolve the heterogeneity and spatial organization of TANs and NET-related states across malignancies. These approaches have identified distinct neutrophil states, spatial defined population, and candidate prognostic or therapeutic biomarkers [134,135,136], while preserving tissue architecture to delineate interactions among neutrophils, tumor cells, stromal cells, and other immune populations [137]. Future studies should integrate single-cell and spatial profiling with multiplex imaging, proteomics, and direct experimental validation of NET formation to distinguish active NETosis from neutrophil abundance alone. Importantly, prospective longitudinal studies are needed to determine whether NET-associated signatures are predictive or treatment response rather than merely prognostic. Neoadjuvant studies incorporating paired pre- and post-treatment tissue specimens may provide a particularly valuable framework for characterizing treatment-induced changes in NET formation, neutrophil states, and tumor–immune interactions. Such integrated approaches may ultimately enable biomarker-guided patient selection and inform rational combinations of NET-targeted therapies with ICIs or other immunotherapies.

## 7. Conclusions

NETosis is emerging as a key link between innate immunity, tumor progression, adverse prognosis, and resistance to cancer immunotherapy. By remodeling the tumor microenvironment, altering immune-cell function, and shielding tumor cells from cytotoxic attack, NETs promote metastasis and are increasingly associated with poor clinical outcomes across multiple tumor types. Key pathways, including PD-L1-positive NETs, IL-8/CXCR1/2 signaling, and the PAD4–CXCR2 axis, provide a strong rationale for combining NET-targeted strategies with ICIs. Future studies integrating standardized NET assays, validated biomarkers, and biomarker-guided clinical trials may enable NET modulation to become a practical strategy for improving treatment response and long-term patient outcomes.

## Figures and Tables

**Table 1:** Cancer-specific NET profiles and clinical implications.

Cancer Type	Key NET Inducers	Dominant NET Mechanism	Functional Consequence	Clinical Association	ICI Implication
Hepatocellular carcinoma (HCC) [87]	IL-33, portal hypertension, TANs	NET-associated cathepsin G component enhances cancer cell invasion	Portal vein thrombus formation; intrahepatic dissemination	NET density inversely correlated with DFS; portal vein invasion marker	High NET burden → immune exclusion; DNase I + PD-L1 co-blockade synergy
Pancreatic ductal adenocarcinoma (PDAC) [21,81]	CAF-derived IL-6, IL-8, TGF-β1; RAGE; HMGB1	Gemcitabine-induced tumor IL-8 secretion drives CXCR1/2-mediated chemoNETosis	Pancreatic stellate cell activation and desmoplasia; chemotherapy resistance	Circulating MPO-DNA elevated in poor chemotherapy responders; NETs accumulate with advanced stage and portal vein invasion	High NET burden limits PD-1/CTLA-4 blockade efficacy in desmoplastic tumors; NET-PAD4 co-targeting under preclinical investigation
Non-small cell lung cancer (NSCLC) [49,89]	CXCL5-recruited TAN (PD-L1^high^); TRAPs; radiotherapy DAMPs	CXCL5-PXN/AKT axis upregulates neutrophil PD-L1	Suppression of CD8^+^ T-cell cytotoxicity; immune evasion	High NET density → shorter PFS	PAD4 KO/JBI-589 + anti-PD-1 synergy
Colorectal cancer (CRC) [13,46]	CXCL5/8; platelet crosstalk; post-hepatectomy inflammatory milieu	NET-driven suppression of CD8^+^ T-cell effector function	CD8^+^ T-cell exhaustion and dysfunction; impaired antitumor immunity in liver metastases	Elevated NETs in CRC liver metastasis associated with T-cell dysfunction and impaired antitumor immunity	NET degradation restores CD8^+^ T-cell function and PD-1 blockade sensitivity in preclinical CRC liver metastasis models
Breast cancer [14,56]	TRAPs; HMGB1; platelet TLR4; CSC-derived IL-8	TRAP-induced formation of PD-L1-decorated NETs via HMGB1-TLR4-MyD88-ERK/p38 axis	Pulmonary pre-metastatic niche formation; NK/T cell suppression	NET density associates with distant metastasis; CSC-NET feedback promotes EMT and CD44^high^ stemness phenotype	DNase I + PD-L1 co-blockade restores function; reduces lung metastasis in preclinical models

**Table 2:** Therapeutic strategies targeting NETosis in cancer immunotherapy.

Category	Agent/Approach	Mechanism	Preclinical Evidence	Clinical Status	Safety Consideration
PAD4 inhibitors	GSK484 [72,110]	Reversible PAD4 inhibitor; suppresses histone citrullination and NET deployment	Prevents NET-induced awakening of dormant cancer cells	Preclinical	Favorable rodent half-life; limited potency restricts clinical translatability
	BMS-P5 [111]	Potent, PAD4-selective histone-citrullination inhibitor	Blocks multiple-myeloma-induced NET formation; delays disease progression in syngeneic mouse model	Preclinical	PAD4-selective pharmacology limits off-target PAD1-3 inhibition relative to pan-PAD agents; clinical safety remains unestablished
	JBI-589 [49]	Selective PAD4 inhibitor; downregulates CXCR2 to block neutrophil trafficking	Synergizes with PD-1/PD-L1 blockade; reduces PMN-MDSC infiltration	Preclinical	No overt toxicity in murine models; clinical safety remains unestablished
DNase I approaches	Systemic DNase I [54]	Enzymatic NET degradation; reduces physical tumor shielding	Reduces physical tumor cell shielding and metastasis in preclinical models; combination with CAR-T remains conceptual, pending direct validation	Preclinical	Systemic immunosuppression risk; infection surveillance required
	LNP-DNase I [112]	Tumor-homing nanoparticles; intratumoral enrichment	Reduces NET density without systemic neutrophil impairment	Preclinical	Host-defense effects and clinical safety remain unestablished
CXCR2 antagonists	Reparixin [46,113]	Block CXCL5/IL8-mediated neutrophil and PMN-MDSC recruitment	IL-8/CXCR2 axis suppression reduces NET density and TAN infiltration	Phase I/II	Acceptable; risk of impairing emergency granulopoiesis
	SB-265610 [114]	Block CXCL5/IL8-CXCR2 signaling to suppress neutrophil activation and NOX-driven NETosis	Reduces tumor-induced NETosis and PMN-MDSC infiltration in murine cancer models	Preclinical	High CXCR2 selectivity limits off-target effects
Complement C3/C5aR antagonists	Avacopan [115]	Block C5a-C5aR1 axis to suppress complement-driven NETosis	C5aR blockade suppresses NETosis and synergizes with anti-PD-1 in PDAC preclinical models	Preclinical	Risk of impairing complement-mediated pathogen clearance
	PMX53 [116,117]	Cyclic peptide C5aR1 antagonist; blocks C5a-driven neutrophil priming upstream of NOX	Reduces NET-dependent pre-metastatic niche formation in murine tumor models	Preclinical	Risk of impairing complement-mediated pathogen clearance
	SB290157 [48,118]	Non-peptide C3aR antagonist; blocks C3a-C3aR signaling that facilitates CR3-mediated NET induction (note: subsequent studies found off-target/partial C5aR2 agonist activity, limiting specificity)	Reduces C3a-driven NETosis and NET-dependent coagulation in tumor models	Preclinical	Risk of impairing complement-mediated pathogen clearance
Platelet TLR4/platelet-neutrophil axis	TAK-242 [119]	Small molecule TLR4 antagonist; disrupts platelet TLR4-mediated vital NETosis by blocking TLR4 signaling adaptor	Reduces NETosis in sepsis and cancer-associated thrombosis models	Phase III trial terminated (insufficient efficacy)	Antiplatelet effects may increase bleeding risk; careful patient selection in thrombocytopenic oncology patients
	Anti-GPVI antibody [120,121]	Blocks GPVI-collagen interaction to prevent platelet activation and platelet-neutrophil complex formation in tumor vasculature	Reduces platelet-neutrophil aggregates and NET levels in murine tumor models	Preclinical	Risk of impairing collagen-dependent platelet hemostasis; monitor for bleeding
	P-selectin blockade (Crizanlizumab) [122,123]	Prevents P-selectin-PSGL-1-mediated platelet-neutrophil adhesion; disrupts tumor vasculature NET amplification loop	Reduces platelet-neutrophil complex formation and NET density in cancer models	Preclinical	Mild antiplatelet effects; generally well-tolerated in clinical use
HMGB1 neutralization	Glycyrrhizin [55,124]	Directly binds HMGB1 to block HMGB1-TLR4/RAGE-ERK/p38 axis and prevent PAD4-dependent NETosis	Reduces NET formation and tumor-associated inflammation in HCC and PDAC models	Preclinical	Risk of hypertension and hypokalemia with chronic use; monitor electrolytes
	Anti-HMGB1 antibody [91,125]	Monoclonal antibody neutralizes extracellular HMGB1; disrupts HMGB1-driven TLR4/RAGE signaling and NET-dependent pre-metastatic niche formation	Reduces NET formation in HCC and PDAC models, and also reduces NET-driven pre-metastatic formation in murine	Preclinical	Well-tolerated in preclinical models
Neutrophil elastase (NE) inhibitors	Sivelestat [126,127]	Blocks NE-mediated GSDMD cleavage, NE nuclear translocation and ECM remodeling	Reduces NET formation and metastasis in murine models	Approved for Acute Lung Injury (ALI) in Japan	Risk of impairing neutrophil bactericidal activity
	AZD9668 [128,129]	Orally bioavailable, reversible, selective NE inhibitor; attenuates NE-driven chromatin decondensation and NET scaffolding	Reduces lung metastasis in breast cancer murine models	Phase II completed in COPD/bronchiectasis	Risk of impairing neutrophil bactericidal activity
Biomaterial platforms	pH-responsive hydrogels [106]	Sustained intratumoral release triggered by acidic, hyperthermic TME	Prolonged NET suppression with single injection; TME-selective	Preclinical	Local delivery minimizes systemic exposure
Combination strategies	NET inhibition + ICI [14,49]	NET degradation enhances CD8^+^ T infiltration, reverses exhaustion, disrupts immune exclusion	DNase I/PAD4 + anti-PD-1 synergy in CRC, PDAC, breast; TRAP-PD-L1 axis reversed	Preclinical	Infection risk during combination; NET biomarker-guided dosing needed

**Table 3:** Clinical studies of NETs in oncology.

Tumor Type	Sample Size	NET Marker(s)	Therapy/Context	Endpoint	Main Result
Multiple advanced cancers	60 patients (observational)	Plasma CitH3	No intervention (biomarker study)	Short-term mortality	Elevated CitH3 independently predicted a 2-fold increase in short-term mortality risk [24]
Multiple cancers	957 patients (prospective, 2-yr follow-up)	CitH3, cell-free DNA, nucleosomes	No intervention (biomarker study)	All-cause mortality	Elevated H3Cit independently associated with increased mortality risk [25]
Cervical cancer	126 patients	Stromal MPO/CitH3 (dual immunofluorescence)	Surgical resection	Recurrence-free survival	High stromal NET density independently predicted recurrence (HR = 2.66), providing prognostic information beyond TAN density alone [26]
Bladder cancer	Retrospective cohort	Serum MPO-DNA; tumor CitH3 positivity	Immune checkpoint inhibitor (ICI) therapy	ICI benefit, metastasis, progression-free survival (PFS)	Elevated MPO-DNA/CitH3 independently associated with reduced ICI benefit, increased metastasis, and shorter PFS [94]
Non-small cell lung cancer (NSCLC)	Retrospective cohort	Serum NET markers + CD8^+^ TIL density + tumor proportion score (composite model)	PD-1 inhibitor therapy	Prediction of treatment response	Composite biomarker model improved prediction of PD-1 inhibitor efficacy compared with single markers [95]
Lung adenocarcinoma	Retrospective cohort	NET-associated long non-coding RNA (lncRNA) signature	Not specified	Prognosis; immune checkpoint gene expression	Signature independently stratified prognosis and correlated with immune checkpoint gene expression [96]
Advanced NSCLC	115 patients	Tumor-associated neutrophil polarization status + NET positivity (multiplex immunofluorescence)	First-line immunotherapy	PFS, overall survival (OS)	NETs-low phenotype associated with improved PFS (15.0 vs. 9.9 months) and OS (40.5 vs. 22.0 months) versus NETs-high [133]

## Data Availability

No new data were created or analyzed in this study. Data sharing is not applicable to this article.

## Abbreviations

The following abbreviations are used in this manuscript:

ACPA	anti-citrullinated protein antibody
AIM2	absent in melanoma 2
AKT	protein kinase B
C3aR	C3a receptor
C5a	complement component 5a
C5aR1	C5a receptor 1
CAF	cancer-associated fibroblast
CAR-T	chimeric antigen receptor T (cell)
CARD	caspase recruitment domain
CD112	cluster of differentiation 112 (nectin-2)
CD155	cluster of differentiation 155 (poliovirus receptor)
CD24	cluster of differentiation 24
CD44	cluster of differentiation 44
CDK4/6	cyclin-dependent kinases 4 and 6
CG	cathepsin G
cGAS–STING	cyclic GMP-AMP synthase–stimulator of interferon genes
CitH3	citrullinated histone H3
CR1/CR3	complement receptors 1 and 3
CRC	colorectal cancer
CSC	cancer stem cell
CT	computed tomography
CTLA-4	cytotoxic T-lymphocyte-associated protein 4
CXCL5	C-X-C motif chemokine ligand 5
CXCR1/2	C-X-C chemokine receptor types 1 and 2
CYBB	cytochrome b-245 beta chain
DAMP	damage-associated molecular pattern
DC	dendritic cell
DNase I	deoxyribonuclease I
dsDNA	double-stranded DNA
ECM	extracellular matrix
EGFR	epidermal growth factor receptor
EMT	epithelial–mesenchymal transition
ERK	extracellular signal-regulated kinase
FcR	Fc receptor
FGL1	fibrinogen-like protein 1
GSDMD	gasdermin D
HCC	hepatocellular carcinoma
HIF-1α	hypoxia-inducible factor 1α
HMGB1	high-mobility group box 1
ICI	immune checkpoint inhibitor
IDO1	indoleamine 2,3-dioxygenase 1
IFN-γ	interferon-γ
IL-15	interleukin-15
IL-18	interleukin-18
IL-1β	interleukin-1β
IL-2	interleukin-2
IL-6	interleukin-6
IL-8	interleukin-8
irAE	immune-related adverse event
iRGD	internalizing arginine-glycine-aspartic acid (RGD) peptide
LAG-3	lymphocyte-activation gene 3
lncRNA	long non-coding RNA
LPS	lipopolysaccharide
MAPK	mitogen-activated protein kinase
MDSC	myeloid-derived suppressor cell
miR-301a-3p	microRNA-301a-3p
miRNA	microRNA
MMP-9	matrix metalloproteinase 9
MPO	myeloperoxidase
mtDNA	mitochondrial DNA
mtNET	mitochondrial DNA-derived neutrophil extracellular trap
mtROS	mitochondrial reactive oxygen species
MyD88	myeloid differentiation primary response 88
NADPH	nicotinamide adenine dinucleotide phosphate
NCF1	neutrophil cytosolic factor 1
NCF2	neutrophil cytosolic factor 2
NE	neutrophil elastase
NET	neutrophil extracellular trap
NETosis	neutrophil extracellular trap formation
NF-κB	nuclear factor-κB
NK	natural killer (cell)
NLRP3	NOD-, LRR- and pyrin domain-containing protein 3
NOX	NADPH oxidase
NPM1	nucleophosmin 1
NSCLC	non-small cell lung cancer
OS	overall survival
ox-mtDNA	oxidized mitochondrial DNA
PAD2	peptidylarginine deiminase 2
PAD4	peptidylarginine deiminase 4
PADI4	peptidylarginine deiminase 4 (gene)
PAF	platelet-activating factor
PD-1	programmed cell death protein 1
PD-L1	programmed death-ligand 1
PDAC	pancreatic ductal adenocarcinoma
PFS	progression-free survival
PGC-1α	peroxisome proliferator-activated receptor-γ coactivator 1α
PMA	phorbol 12-myristate 13-acetate
PMN-MDSC	polymorphonuclear myeloid-derived suppressor cell
PVR	poliovirus receptor (CD155)
PVTT	portal vein tumor thrombosis
PXN	paxillin
RAGE	receptor for advanced glycation end products
RFS/DFS	recurrence-free/disease-free survival
ROS	reactive oxygen species
SK	small-conductance calcium-activated potassium (channel)
SK3	small-conductance calcium-activated potassium channel 3
SNAI1	Snail family transcriptional repressor 1
SNAI2	Snail family transcriptional repressor 2
STAT3	signal transducer and activator of transcription 3
TAM	tumor-associated macrophage
TAN	tumor-associated neutrophil
TGF-β	transforming growth factor-β
Th17	T helper 17 (cell)
TIGIT	T cell immunoreceptor with Ig and ITIM domains
TIM-3	T-cell immunoglobulin and mucin-domain-containing protein 3
TLR	Toll-like receptor
TLS	tertiary lymphoid structure
TME	tumor microenvironment
TNF-α	tumor necrosis factor-α
TPS	tumor proportion score
TRAP	tumor cell-released autophagosome
Treg	regulatory T cell
TXA2	thromboxane A2
ZEB1	zinc finger E-box-binding homeobox 1
